# 434. Reduced Gut Microbiome Diversity and Compositional Characteristics are Associated with Postoperative Infection in Heart Transplant Recipients

**DOI:** 10.1093/ofid/ofae631.148

**Published:** 2025-01-29

**Authors:** Christopher Kaperak, Eric Roessler, Huaiying Lin, Mark Dela Cruz, Emerald Adler, Jackelyn Cantoral, Caroline Jadczak, Elizabeth Bell, Eric Pamer, Christopher Lehmann, Ann Nguyen

**Affiliations:** University of Chicago Medicine, Chicago, IL; University of Chicago, Chicago, Illinois; University of Chicago, Chicago, Illinois; Advocate Christ Medical Center, Chicago, Illinois; University of Chicago, Chicago, Illinois; University of Chicago, Chicago, Illinois; University of Chicago, Chicago, Illinois; University of Chicago Medicine, Chicago, IL; University of Chicago, Chicago, Illinois; University of Chicago, Chicago, Illinois; University of Chicago, Chicago, Illinois

## Abstract

**Background:**

The human microbiome has been linked to important clinical outcomes, including postoperative infection. Loss of diversity has been associated with drug resistant organism colonization, infection, immune defenses, epithelial barrier integrity, and death. The microbiome’s role in postoperative infection among heart transplant (HT) recipients remains poorly understood.

**Table 1:**
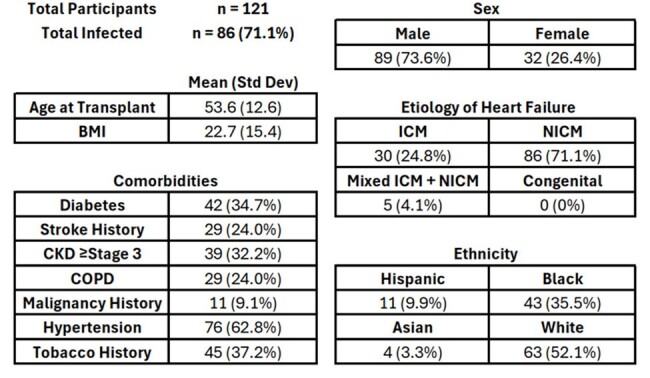
Clinical Characteristics Table 1 Clinical Characteristics: Characteristics noted by number and percent or mean and Standard Deviation where appropriate. Abbreviations: ICM – Ischemic Cardiomyopathy; NICM – Non-ischemic cardiomyopathy; CKD – Chronic Kidney Disease; COPD – Chronic Obstructive Pulmonary Disease.

**Methods:**

Stool microbiomes for 121 HT recipients were determined by metagenomic sequencing. Infections occurring in the first 100 days following transplant were aggregated. Infection was defined as the presence of a compatible clinical syndrome and a positive test result via culture, PCR, serology, or imaging. To determine if infection risk was related to stool microbiome composition, samples at the time of transplant were shotgun sequenced and taxonomy was determined using MetaPhLan 4. Alpha diversity was measured by inverse Simpson, beta diversity was measure by Bray-Curtis dissimilarity.

**Table 2:**
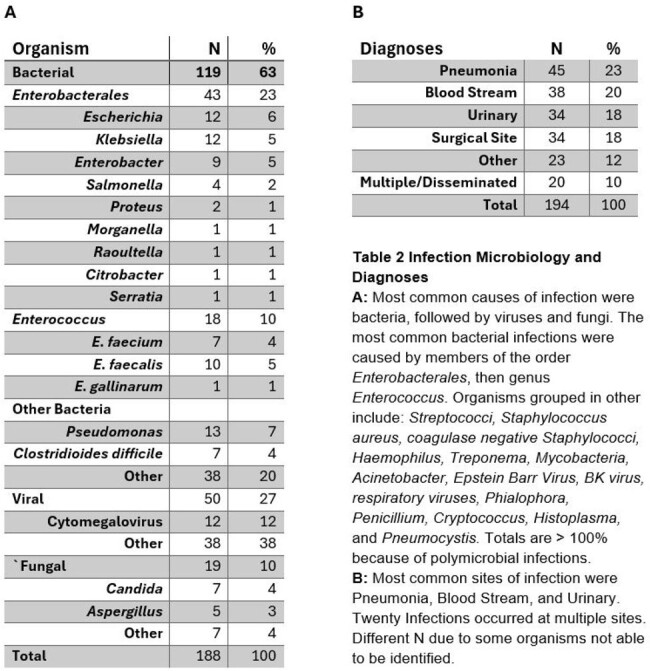
Infection Microbiology and Diagnoses

**Results:**

Average age at HT was 53.6 years and 73% were male, and the cause of heart failure in 71% was nonischemic cardiomyopathy. 194 infections occurred among 72 (60%) patients (Table 2). The most common infections were pneumonia, bloodstream, surgical site, and urinary. Most infections were caused by bacteria, specifically *Enterobacerales* and *Enterococci*. (Table 2) Stool microbial Alpha diversity was lower in patients who developed infection; p=0.0026. (Figure 1) Stool microbiome composition also differed significantly between groups. Patients with postoperative infection experienced more single species expansions, most notably *Enterococcus* and *Enterobacterales*, which were common etiologies of infection. (Figure 2) Uninfected patients had more abundant obligate anaerobic taxa including Bacteroidetes, *Lachnospiraceae*, and *Ruminococcaceae*.

**Figure 1:**
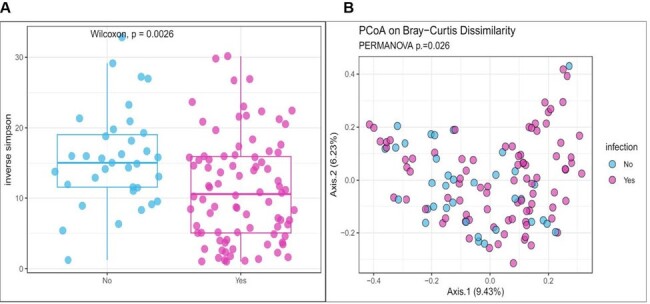
Alpha and Beta Diversity of Stool Microbiota: A. Stool microbiome alpha-diversity by inverse Simpson p= 0.0026 Wilcoxon. B. Stool microbiome beta-diversity by PcoA Bray-Curtis Dissimilarity p=0.026 PERMANOVA.

**Conclusion:**

The stool microbiome of HT patients with postoperative infection is marked by lower alpha diversity and notable compositional differences including *Enterococci* and *Enterobacterales* expansion coupled with reduced Bacteroidetes, *Lachnospiraceae*, and *Ruminococcaceae*. Further study into the interaction between these organisms and the host are needed to better understand their role in infection.

**Figure:**
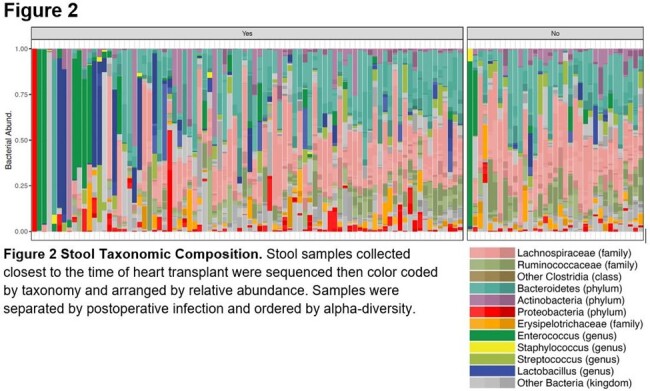

**Disclosures:**

**All Authors**: No reported disclosures

